# Roaming the Neighbourhood: Influences of Independent Mobility Parenting Practices and Parental Perceived Environment on Children’s Territorial Range

**DOI:** 10.3390/ijerph16173129

**Published:** 2019-08-28

**Authors:** Janae Vlaar, Mariana Brussoni, Ian Janssen, Louise C. Mâsse

**Affiliations:** 1 School of Population and Public Health, University of British Columbia, 2329 West Mall, Vancouver, BC V6T 1Z4, Canada; 2 British Columbia Children’s Hospital Research Institute, 4480 Oak St., Vancouver, BC V6H 3N1, Canada; 3 Department of Pediatrics, University of British Columbia, 2329 West Mall, Vancouver, BC V6T 1Z4, Canada; 4 School of Kinesiology and Health Studies, Queen’s University, 99 University Ave., Kingston, ON K7L 3N6, Canada

**Keywords:** independent mobility, activity spaces, neighbourhood activity

## Abstract

Children’s independent mobility (IM), their freedom to move about their neighbourhood without supervision by adults, has been in steady decline in recent decades. Previous research has linked perceptions of the environment with various measures of IM, but recently concerns have been raised regarding inconsistency in measuring IM. This study used various measures of IM and aimed to address how parental perceptions of the neighbourhood environment are associated with children’s territorial range (actual IM), as well as how this relationship is mediated by IM parenting practices (allowed IM). A sample of 105 child/parent dyads from Vancouver, Canada participated in this study. Children (age 10–13) wore a global positioning system (GPS) watch and an accelerometer and completed an activity diary for seven days to assess their territorial range. Parents completed a questionnaire that assessed perceptions of their neighbourhood environment and IM parenting practices—license for IM and roaming allowance. Path analyses were used to address the research aims. License for IM and roaming allowance mediated the relationship between perceived walking facilities, crime safety, and neighbourhood relations and children’s territorial range. Findings suggest that future interventions to increase children’s territorial range should focus primarily on attitude and behaviour change among parents to grant children more freedom.

## 1. Introduction

Independent mobility (IM)—the freedom that children have to be in and move about their neighbourhood without adult supervision, either on their own or accompanied by peers [1,2,3,4]—has markedly declined in recent decades [1,3,5,6,7]. This is not for lack of importance to child development and well-being; IM provides opportunities for children to engage in active transportation and outdoor play which in turn promote accumulation of physical activity (PA), interaction with their environment, development of cognitive skills, and social interaction [6,8,9,10,11,12,13,14]. These opportunities that IM affords are important to many aspects of children’s health and well-being: prevention of chronic physical health issues [8]; improved adaptability, stress regulation, and executive functioning [9], as well as risk-management and problem-solving [11,15,16]; enhanced spatial awareness, navigation, and way-finding skills [15]; and enriched cooperation and social relationships [10,11]. 

Socioecological models for active living [17] and health behaviours [18] provide a useful framework for examining these influences. A number of proximal factors, such as intrapersonal, interpersonal, and neighbourhood and community factors, influence children’s IM to varying degrees [4,7,19,20,21,22,23,24,25,26]. Intrapersonal factors, such as child age and gender, have been studied extensively, leading to the general conclusion that children who are older and male have greater IM than children who are younger and female [4,19,20]. Interpersonal factors, specifically parenting practices related to IM, are important to consider when examining children’s IM. Parents act as “gatekeepers” to their children’s activities [27] and the permissions or restrictions they apply may influence their children’s IM [21]. In addition, neighbourhood environment factors, particularly parental perceptual factors relating to the neighbourhood environment, have been found to be associated with children’s IM [22,23]. The strongest evidence for the association of perceptual factors with children’s IM has been perceptions of safety of the physical and social environments [7,19,24,25]; however, perceived access to walking or cycling facilities has also been associated with children’s IM [22,28].

While there has been a growing body of research on how children move in and around their neighbourhoods without adult supervision, there is a lack of consensus about how IM is specifically defined and measured [29,30]. This is problematic because different measures consider different aspects of IM. For example, the license for IM scale is an indicator of restrictions or permissions for children’s IM. License for IM indicates whether or not a child is allowed to do certain things without supervision, such as crossing a main road or travelling to school, providing certain behavioural boundaries [3]. This is in contrast to subjective spatial measures which assess how far from home a child can go without supervision [31] and indicates whether or not a child is allowed to go certain places without supervision. Finally, objective spatial measures use global positioning system (GPS) devices to track children’s actual IM [32,33,34]. Bhosale et al. suggest that IM measures are unidimensional and may not give a complete understanding of children’s IM [29]. Few studies have examined children’s objectively measured IM [32,33,34], often using self- or parent-reported data instead [29,35], which assumes that children’s actual IM reflects what they or their parents report. This suggests the importance of distinguishing between the degree of allowed IM and actual IM, as children do not necessarily obey parent-imposed IM restrictions or permissions for either behavioural or spatial IM [36]. This study conceptualizes IM parenting practices as making up a child’s allowed IM; both the things and places children are given permission to do or access on their own (e.g., license for IM [1,3] and roaming allowance [31]). The term territorial range is used to conceptualize children’s actual IM (referred to as activity spaces, home ranges, activity ranges, or IM area in previous literature [34]).

**Figure 1:**
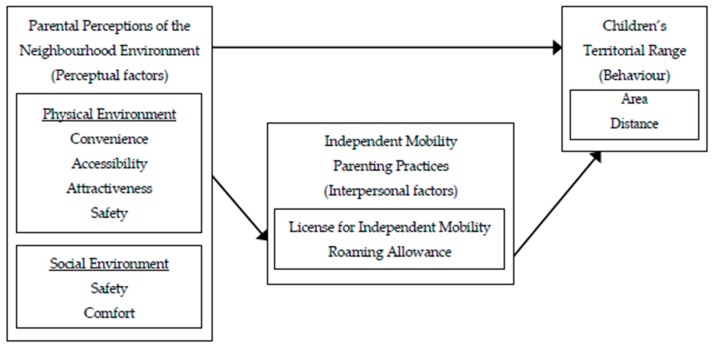
Theoretical model examining how parental perceptions of the neighbourhood environment relate to children’s territorial range and mediation by independent mobility parenting practices.

Therefore, the purpose of this study was to examine the association between parental perceptions of the neighbourhood environment and different measures of IM. This study considered both the direct association of parental perceptions of the neighbourhood environment as well as the mediating role of IM parenting practices on children’s territorial range to provide a more in depth understanding of the relationship between allowed IM and actual IM, and how the neighbourhood influences it. Figure 1 presents the conceptual relationships between parental perceptions of the neighbourhood environment, IM parenting practices (e.g., license for IM, roaming allowance), and children’s territorial range examined in this study.

## 2. Materials and Methods 

### 2.1. Study Design, Setting, and Sample

This study used data from “The state of play: Socio-ecological perspectives on children’s outdoor play”, a mixed-methods study of children’s outdoor play related to neighbourhood design in Vancouver, British Columbia [37]. Data were collected from 2016 to 2018 in April, May, June, September, and October. This data collection period allowed the effects of school and weather to be controlled for by avoiding collecting data during the rainy season with less daylight hours or during the summer break when daily routines change. The study was conducted in three neighbourhoods with varying environmental characteristics (e.g., population density, street network typology, green spaces, residential density): Grandview-Woodland, Steveston, and Lonsdale. 

Children aged 10 to 13 and their parent(s) were recruited from different neighbourhoods in Metro Vancouver. Sampling was purposeful to include children with varying degrees of allowed IM and include an even distribution of age and gender among the child participants. To be eligible, children had to have English literacy and reside in one of the three catchment areas. Children were excluded if they had any disability or health condition that limited their participation in PA, if they had psychiatric problems, or if they required learning assistance. Additionally, children had to spend their entire study week in one home (e.g., no sleep overs). Families were screened for level of IM through the question: In the past months, how far has your child roamed on her/his own (or with friends)? Each participating child received an honorarium of $100 total over two meetings and participating parents received an honorarium of $50 after the second meeting as recognition for their time and effort to participate. 

This study was approved by the Behavioural Research Ethics Board at the University of British Columbia (#H15-02190). Informed consent and assent were obtained from each participating parent and child. 

A full description of the data collection procedures is available elsewhere [37]. In summary, after being screened for eligibility, each participating family had a meeting with a research assistant at a mutually agreeable time in the family’s home or another agreed upon location. At the initial meeting, children and parents were again verbally briefed on the study purpose and procedures and gave written assent and consent, respectively. Parents were given daily surveys about their children’s unsupervised activities and children were fitted with GPS watches (Garmin Forerunner 220) and accelerometers (Actical, Philips Respironics, Bend OR) and taught how to wear the devices. Children were instructed to wear the GPS watch on their wrist, making sure to save the data at the end of each day and restart the GPS as soon as they woke up each morning. Children were also given daily activity diaries to complete, indicating start and end times of where they were, who they were with, what they were doing throughout the day, and when they removed the devices. Data were collected for seven days. At the end of the data collection period the devices, children’s daily activity diaries, parent daily surveys, and both child and parent demographic questionnaires were picked up by research study personnel and processed to allow children to review their mobility data at a follow-up meeting.

The GPS and accelerometer data were uploaded, merged, and coded using Personal Activity and Location Measurement System (PALMS) software (University of California San Francisco, Release 1.4.2). The end product of this merge was a file for each participant that contained 40,320 time-stamped rows of data (one row for each 15-second epoch over a seven day measurement period), and several columns that included the time of the measure, the accelerometer count value, and the longitude and latitude coordinates recoded by the GPS watch. As explained below, additional columns of data were entered into this file, first in an automated manner by PALMS and then manually by the research team. PALMS is a web-based integrated measurement system that is capable of merging and processing time-stamped PA data collected with accelerometers and location data collected with GPS devices [38]. PALMS software contains validated algorithms that used the GPS data to identify rows of data that occurred during a trip and, in a separate column, the transportation mode (walking, bicycling, or vehicle) of each trip [38].

The merged output was manually cleaned by one of five research assistants. Missing GPS coordinates were identified and, when possible, GPS coordinates were imputed using children’s daily activity diaries and parents’ daily surveys. Any coordinates that remained missing were noted. The activity diaries were then used to confirm the trip numbers and transportation modes that PALMS calculated and any discrepancies were noted. Columns of data that reflected indoor/outdoor location (inside, outside, both), accompaniment (alone, with friends or siblings, with adults), and activity type (verbatim of child written description) were inputted based on children’s daily surveys. This resulted in the creation of a seven-day location and activity profile for each child. Any gaps in this information were noted and addressed at the follow-up meeting to finish cleaning the data and prepare a finalized data set.

### 2.2. Measures 

Territorial range: Territorial range was assessed using the child’s seven-day location and activity profile. Children’s territorial range was split into area and distance and calculated in four stages: 1) identifying routes travelled and destinations accessed independently by each child, 2) delineating a boundary for each child’s territorial range, 3) calculating the area of the territorial range, and 4) calculating the furthest straight line distance of the territorial range. The seven-day location and activity profiles for each participant were imported into ArcGIS (ArcGIS Version 10.5, Environmental Systems Research Institute (ESRI), Redlands, CA, USA) for further processing. GPS coordinates were selected by attribute for times when the child was outside or on public transit and unsupervised (alone or with friends/siblings), resulting in a seven-day territorial range profile for each child. The seven-day territorial range map was visually inspected for any trips or activities that did not connect to the participant’s home location. Trips that did not connect to the participant’s home location were manually removed, as they were not representative of places the child could access independently from home. The final result was a clean seven-day territorial range from home profile for each child. Convex hull geometry was then used to enclose the child’s territorial range in the smallest possible polygon. Convex hull geometry is a common method of defining children’s activity spaces [39,40,41]. Territorial range area was calculated as the space within the polygon and territorial range distance was calculated as the maximum straight-line distance from home to the furthest edge or vertex of the polygon.

Neighbourhood Environment: Parental perceptions of the physical environment were assessed using the Neighbourhood Environment Walkability Scale (NEWS) parent-report youth version [42]. The different aspects of the physical environment assessed were: destinations (20 items, six response options), recreation facilities (14 items, six response options), residential density (four items, five response options), land-use mix accessibility (five items, four response options), street connectivity (three items, four response options), walking facilities (three items, four response options), aesthetics (four items, four response options), and traffic safety (seven items, four response options) [42]. In a sample of parents of children and adolescents from the US, the internal consistency of the scale ranged from acceptable to excellent (α = 0.72 to α = 0.93) and the test–retest reliability ranged from moderate to adequate (intraclass correlation coefficient (ICC )= 0.61 to ICC = 0.82) [42].

Parental perceptions of the social environment were assessed with three questionnaires. The NEWS crime scale (six items, four response options) was used to assess perceived stranger danger (e.g., “I am worried about letting my child play outside alone around my home because I am afraid of them being taken or hurt by a stranger”) and had good internal consistency (α = 0.81 to α = 0.87) and adequate test–retest reliability (ICC = 0.78 to ICC = 0.87) in a sample of parents of adolescents from the U.S. [42]. The Social Danger Perception Scale (seven items, four response options) was used to assess perceived micro-crimes and social incivilities (e.g., “In the streets around my home, there are neglected areas (dirty, with large abandoned objects, etc.)”) and had good internal consistency (α = 0.83) in a sample of parents of Italian school-aged children [43]. The Neighbourhood Relations Scale (five items, five response options) was used to assess perceived social cohesion and capital within the neighbourhood (e.g., “I exchange favours with my neighbours”) and the scale on which it was based had good internal consistency (α = 0.86 to α = 0.89) in a sample of parents of Italian children [24].

IM Parenting Practices: IM parenting practices were assessed using two questionnaires about children’s independent mobility: license for IM and roaming allowance. The license for IM scale (subjective behavioural) includes six yes/no questions that ask parents whether they allow their child to do certain things: cross main roads alone, travel to places other than school within walking distance alone, travel home from school alone, go out alone after dark, cycle on main roads alone, and use local buses alone [1]. Licenses are conceptualized to be granted to children by their parents as recognition that they are competent enough to do certain things within the context of their neighbourhood. The number of licenses a parent grants a child represents the degree of IM a parent allows their child to have [3]. An index for each child was calculated from the six mobility licenses. The license for IM scale had adequate test–retest reliability (ICC = 0.83) in a sample of parents of Canadian elementary school children [44].

Children’s roaming allowance (subjective spatial) was determined through parent-report on the parent demographic questionnaire. Based on a question developed by Veitch et al. parents were asked: How far is your child allowed to roam on his/her own (or with friends) without adult accompaniment [31]? Response options were: “My child is not allowed out alone”, “my child is allowed out within my yard and/or driveway”, “my child is allowed out within my street”, “my child is allowed out within two to three streets from my home”, “my child is allowed out within a 15 minute walk from home”, “my child is allowed out more than a 15 minute walk from home” [31]. Responses were coded from one to six, with high scores representing a large roaming allowance. Test–retest reliability for this scale was moderate (κ = 0.52 to κ = 0.59) [31].

Participant characteristics: Individual socio-demographic data were collected for each parent and child participant. Children reported their age, gender, grade, and race. Parents reported their child’s age and gender and their own age, gender, race, marital status, education, employment status, household income, household dwelling type, and number of kids. 

### 2.3. Analysis

Descriptive statistics were presented as percentage or mean, standard deviation (SD), and range. 

Path analyses was conducted to examine the direct effect of independent variables (parental perceptions of the neighbourhood environment) on the dependent variables (children’s territorial range area and distance), as well as indirect effects through potential mediating variables (license for IM, roaming allowance). Child age, child gender, and household income were included as covariates in all models. Outcome variables were log transformed to account for non-normal distributions. Standardized coefficients were calculated to account for measurement on different scales. Model parameters were estimated using full information maximum likelihood estimation. Significance was set at *p* < 0.05. In total, four models were run (Table 1). All analyses were completed using Stata 15 (StataCorp, College Station, TX, USA).

## 3. Results

### 3.1. Demographic Characteristics

The final sample included 105 children and 135 parents. For the 30 children who had two participating parents, the data for the parent who initially contacted the study team was used. Parents who were not included in the study were significantly more likely to be male, but did not differ from included parents by other socio-demographic characteristics, IM parenting practices scores, or any of the perceived neighbourhood environment variables. 

The demographic characteristics of the sample of parent–child dyads (N = 105) are in Table 2. There was an even distribution of child participants among the three catchment areas. The children were equally split by age and gender. The majority of participating parents were mothers (85.7%). Participating families were equally distributed among the three neighbourhoods. The sample was predominantly white, well educated, high income, and living in single detached homes. 

### 3.2. Descriptive Statistics for Independent, Dependent, and Mediating Variables 

The distributions of parental perceptions of the environment, IM parenting practices, and children’s territorial range variables are in Table 3. Average scores indicate that parental perceptions of the neighbourhood physical and social environment were high (between three and four on a four-point scale), meaning that parents generally perceived their neighbourhood environment in a positive way (e.g., good walking facilities, good traffic safety, aesthetically pleasing, well connected, places to go). Perceived traffic safety received the lowest score. 

Parents indicated an average roaming allowance of five on a scale of one to six. The average license for IM score was three on a scale of zero to six. This highlighted the discrepancy between subjective spatial measures of IM and subjective behavioural measures of IM; parents in this sample generally allowed their children to go relatively far from home without an adult, but placed restrictions on things they can do without an adult. Overall, territorial range was low, but highly variable. Half of the participants went no further than approximately 800 metres from home, but ranged from not going anywhere to a maximum distance of 32 kilometres. The same was observed for area. Half of the participants stayed within 0.3 square kilometres of their home, but there was a range from some children not going anywhere to children travelling more than 500 square kilometres around their home. It is important to recognize that children who had very high territorial range were outliers, but were real data points and were considered in analysis.

### 3.3. Path Analysis

**Figure 2:**
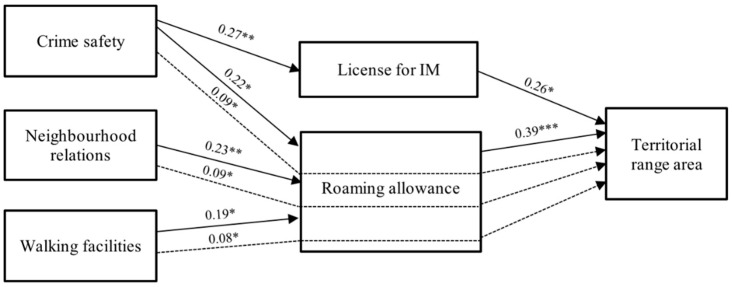
Summary of pathways for significant direct and indirect effects for territorial range area. All path coefficients were standardized. Solid lines indicate direct effects, dashed lines indicate indirect effects. *p < 0.05, ** *p* < 0.01, *** *p* < 0.001; IM: independent mobility.

**Figure 3:**
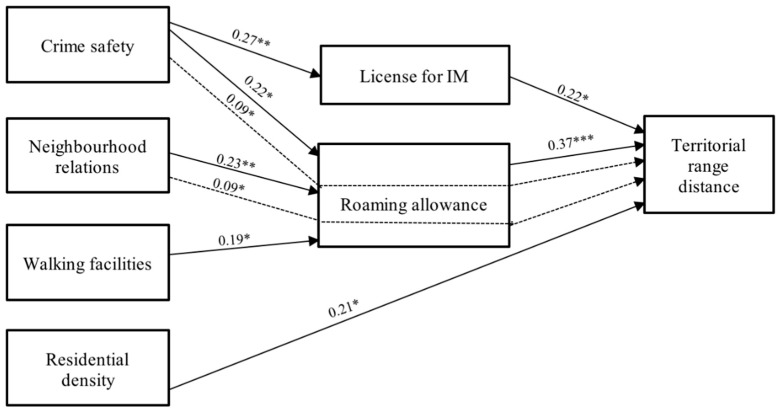
Summary of pathways for significant direct and indirect effects for territorial range distance. All path coefficients were standardized. Solid lines indicate direct effects, dashed lines indicate indirect effects. * *p* < 0.05, ** *p* < 0.01, *** *p* < 0.001; IM: independent mobility.

Results from path analyses are presented in this section. The direct and indirect effects of parental perceptions of the neighbourhood environment on territorial range area and distance are presented in Table 4 and Table 5, respectively. Figure 2 summarizes the significant paths from Table 4 and Figure 3 summarizes the significant paths from Table 5. 

Highlights of the analysis include: a) few significant associations between the parental perceived neighbourhood environment and IM parenting practices (i.e., direct effects between independent variables (IV) and mediating variables (MV)), b) significant association of IM parenting practices with children’s territorial range (i.e., direct effects between MV and dependent variables (DV)), c) only one significant direct association of the parental perceived neighbourhood environment with children’s territorial range (i.e., direct effects between IV and DV), and d) some significant associations between perceived neighbourhood environment and children’s territorial range, mediated by IM parenting practices (i.e., indirect effects between IV and DV) (Figure 2 and Figure 3).

## 4. Discussion

The significant path coefficients between independent and mediating variables and between mediating and dependent variables were all in the positive direction. More positive parental perceptions of crime safety, neighbourhood relations, and walking facilities were found to be significantly associated with increased roaming allowance. More positive parental perceptions of crime safety were also significantly associated with increased license for IM. These results were consistent with existing literature which highlights that perceptions of crime are significantly associated with parenting practices related to IM [1,3,45]. The measure of perceived crime used in this study captures perceived “stranger danger” which has been highlighted throughout the literature as being associated with more restrictive IM parenting practices [1,3,45]. Findings that more positive perceptions of neighbourhood relations were significantly associated with license for IM and roaming allowance were consistent with previous research that indicates that parents who perceive higher social cohesion are more likely to allow children to roam further [46]. The lack of significant association between social danger perception and IM parenting practices was also consistent with previous literature that has found no association between social incivilities (drugs, strange people, neglected areas, etc.) and allowed IM [47].

Greater roaming allowance and license for IM were both significantly associated with increased territorial range area and distance. Roaming allowance showed relatively large positive associations with children’s territorial range, which was somewhat surprising given that it was assessed using a single question. This suggests that asking parents how far they allow their children to go on a scale is representative of how far children actually go in this sample. Roaming allowance may show a strong association with children’s territorial range because it approximates how far the parent allows their child to go, allowing for a high degree of flexibility, but still measuring a spatial aspect of IM. License for IM was also positively associated with territorial range, aligning with existing literature [29], but had a smaller magnitude of association with territorial range than roaming allowance did. One possible explanation for this is that while parental roaming allowance is a spatial measure, license for IM is more of a behavioural measure. It asks about what children are allowed to do, not where they are allowed to go. These results provide further evidence for previous suggestions that license for IM may not accurately reflect children’s actual IM [29].

The sole direct association of parental perceptions of the neighbourhood environment on children’s territorial range was a positive relationship between residential density and territorial range distance. Indirect associations between perceived walking facilities, crime safety, and neighbourhood relations with territorial range, through license for IM and roaming allowance, were observed. This indicated that the license for IM and roaming allowance that parents grant their children mediated the effects of parental perceptions of the neighbourhood environment on children’s territorial range, but only for perceived walking facilities, crime safety, and neighbourhood relations. While the direct effects of perceived walking facilities, crime safety, and neighbourhood relations on territorial range were negative (parents perceive more walking facilities, less stranger danger, or better neighbourhood cohesion, but children go less far from home), when license for IM or roaming allowance were included as mediators (both have positive associations with perceived walking facilities, crime safety, and neighbourhood relations), the sign of the indirect effect flipped, indicating inconsistent mediation.

The negative (but not significant) direct effect between perceived crime safety and territorial range at first seemed counter intuitive and was contradictory to previous findings [7,24,48]: when parents perceive their neighbourhood to be less safe in terms of crime, children’s territorial range is greater. Some possible explanations are that children do not share this fear of stranger danger with their parent(s) because it is less tangible and therefore does not directly influence their territorial range in the expected direction, or that parents are more aware of the danger because they have been to these areas before allowing their children to roam there. Alternatively, parents whose children roam further from home could experience a greater fear of crime because their child is further away (reverse direction of effect), or children of parents who perceive their neighbourhood to be more safe may not have as large of a territorial range because they have friends and things to do close by.

Similarly, more positive perceptions of neighbourhood relations were negatively (but not significantly) associated with territorial range. Previous research by Lin et al. has shown that more positive perceptions of neighbourhood cohesion (willingness to help, neighbours watch out for kids, trust, reciprocity, etc.), and neighbourhood connection (adults know the local children, parents know each other, etc.) were associated with increased number of IM trips among children [49]. One explanation for the negative association seen in this direct path could be that if children have friends on their street and parents perceive this as strong neighbourhood relations, the children do not need to travel as far to play or hang out with their peers.

In summary, parental perceptions of crime safety, neighbourhood relations, and walking facilities were positively associated with IM parenting practices and IM parenting practices were positively associated with territorial range. While the only direct association between parental perceptions of the neighbourhood environment and children’s territorial range was between residential density and distance, the indirect effects of perceived crime safety, neighbourhood relations, and walking facilities on territorial range were positive through IM parenting practices. This exemplified the role of IM parenting practices as a mediator in the relationship between parental perceptions of the neighbourhood environment and territorial range and suggests that future interventions designed to increase children’s IM should focus their attention on changing attitudes among parents to shift towards less restrictive IM parenting practices. 

### 4.1. Study Strengths

To our knowledge, this was the first study to look at parenting practices related to IM as a mediator between parental perceptions of the neighbourhood environment and children’s territorial range. Given that previous research has shown relationships between parental perceptions of the neighbourhood environment and children’s IM and related behaviours, such as active transportation and physical activity [7,19,22,24,25,45,47,50,51,52,53,54] as well as parents’ role in allowing or restricting their children’s IM [5,27,55], it seems important to understand if parenting practices are in the pathway between how they perceive the environment and how far from home their child goes. 

This study included territorial range as an objective measure of children’s IM, derived from a combination of GPS and daily diary data, as opposed to more subjective measures, such as self-report or proxy-report, more commonly used in previous research. The issues with subjective measures are twofold: 1) if self-reported by children, the measures are at risk of recall bias; and 2) if reported by a parent, it assumes that parents know exactly where and what their child is doing, rather than recognizing children as social agents who make their own decisions about where they go and what they do there [30,36,56]. Although this study did use self-reported daily activity diaries, all entries were cross-referenced with the GPS data and any discrepancies were clarified in a follow-up interview. This method of determining territorial range allows the collection of objective location data while still privileging children’s reports, decreasing subjectivity and increasing completeness.

This study also included typically used subjective measures of IM—license for IM and roaming allowance—creating a more complete picture of how these measures are related and what factors are associated with children’s IM. Previous research has typically considered license for IM and roaming allowance as measures of children’s IM. In this study, they were considered as measures of allowed IM and the objective territorial range measurement as actual IM. This offers a unique perspective on the role of these different measurements and what they actually represent, rather than what they are typically used to represent. It also allowed examination of how well different allowed IM measures predict children’s actual IM, clarifying which measure is most closely associated with children’s actual IM. 

### 4.2. Study Limitations

This study was not without its limitations. The primary limitation of the study was that territorial range was derived from cross-sectional data collected over a seven-day period. Therefore, results only captured a small snapshot of children’s potential territorial range and may have resulted in measurement error. For example, a child with a very small territorial range in our study may not have gone places he or she went the week before or will go the week after, making their range seem smaller than reality. In a similar study by Bhosale et al. in which an online application was used by children to map their IM destinations, children’s seven-day IM destination area (median = 0.002 km^2^) was much smaller than their six-month IM destination area (median = 0.160 km^2^), suggesting that in the present study, longer term territorial range may have been underestimated in the present study [29]. Additionally, objective environmental data was not used and children’s perspectives of their neighbourhood environment were not considered. In order to address these limitations, future research should utilize objective environmental assessments and needs to consider the perceived environment from the child’s perspective. Most research on environments and IM privileges adult perceptions, and children see the world in a fundamentally different way [57,58], which may yield different and interesting insights into how children’s territorial range is shaped. Additionally, future studies should verify these findings with larger sample sizes in different geographic locations, as Metro Vancouver has a unique urban environment including low residential density within Vancouver proper compared to other cities of similar size as well as mixed land use in suburban areas. 

## 5. Conclusions

This study conceptualized IM in two distinct ways: allowed IM, indicated by IM parenting practices (license for IM and roaming allowance); and actual IM, indicated by territorial range. This study found that parental perceptions of the neighbourhood environment—specifically crime safety, neighbourhood relations, and walking facilities—were associated with license for IM and roaming allowance to varying degrees. Similarly, license for IM and roaming allowance were associated with territorial range to varying degrees. IM parenting practices were found to mediate the relationship between parental perceptions of the neighbourhood environment and children’s territorial range, suggesting that future interventions to increase children’s actual IM focus on shifting parental attitudes and practices related to allowed IM.

## Figures and Tables

**Table 1:** Conceptual models for path analyses.

Model	Independent Variable	Mediating Variable	Dependent Variable
1	Parental perceptions of neighbourhood environment	License for IM	Territorial range area
2	Parental perceptions of neighbourhood environment	Roaming allowance	Territorial range area
3	Parental perceptions of neighbourhood environment	License for IM	Territorial range distance
4	Parental perceptions of neighbourhood environment	Roaming allowance	Territorial range distance

Note: IM = independent mobility.

**Table 2:** Demographic Characteristics (N = 105).

Variable	% or Mean ± SD (Range)
Child Gender	
Female	50.5
Male	49.5
Child Age	
10	24.8
11	26.7
12	24.8
13	23.8
Child Grade	6 ± 1 (4–9)
Child Race	
White	65.7
Asian	23.8
Other	9.5
Prefer not to answer	1.0
Parent Gender	
Female	85.7
Male	14.3
Parent Age	45 ± 5 (35–56)
Parent Race	
White	70.5
Asian	21.0
Other	7.6
Prefer not to answer	1.0
Parent Marital Status	
Married/Common-law	90.5
Single/Separated/Divorced/Widowed	9.5
Parent Education	
High school or less	10.5
College diploma/trade certificate	23.8
Bachelor’s degree	37.1
Above Bachelor’s degree	28.6
Parent Employment Status	
Employed	79.0
Unemployed	21.0
Household Income	
<$50,000	10.5
$50,000–$89,999	19.0
$90,000–$129,999	24.8
≥$130,000	32.4
Prefer not to answer	13.3
Household Dwelling Type	
Single Detached	57.1
Semi-Detached/Duplex	16.2
Rowhouse/Townhouse	15.2
Apartment	11.4
Children in Household	
1	21.0
2	53.3
3	20.0
4+	5.7

Note: SD = standard deviation; % = percentage. If % do not add up to 100%, it is due to rounding.

**Table 3:** Descriptive statistics for parental perceptions of the neighbourhood environment, IM parenting practices, and children’s territorial range (N = 105).

Variable	Description	Observed Range	Median	Mean ± SD
**Independent Variables**				
Destinations	Perceived availability and variety of shops, services, and food outlets coded from 1 (>30 minutes) to 5 (1–5 minutes).	1.7–5	3.7	3.6 ± 0.8
Recreation Facilities	Perceived availability and variety of parks, gyms, and recreation centres coded from 1 (>30 minutes) to 5 (1–5 minutes).	1.3–4.7	3.4	3.4 ± 0.6
Residential Density	Perceived proportion of different dwelling types, coded from 1 (none) to 5 (all) then scaled based on housing density and summed (40–200).	44–160	120	114 ± 32
Land-use Mix Access	Perceived accessibility of different places coded from 1 (strongly disagree) to 4 (strongly agree), with barriers (e.g., parking, hills, railroad) being reverse coded.	2.5–4	3.5	3.5 ± 0.4
Street Connectivity	Perceived street network typology (e.g., cul-de-sacs, block size, route availability) coded from 1 (strongly disagree) to 4 (strongly agree).	2–4	3.3	3.3 ± 0.5
Walking Facilities	Perceived pedestrian environment quality coded from 1 (strongly disagree) to 4 (strongly agree).	1–4	3.3	3.3 ± 0.7
Aesthetics	Perceived attractiveness of neighbourhood (e.g., trees, nice buildings) coded from 1 (strongly disagree) to 4 (strongly agree).	1–4	3.8	3.5 ± 0.6
Traffic Safety	Perceived safety from traffic (e.g., slow speeds, lighting, crosswalks) coded from 1 (strongly disagree) to 4 (strongly agree). Unsafe features (e.g., speeding, exhaust) were reverse coded.	1.7–3.9	2.9	2.9 ± 0.5
Crime Safety	Perceived stranger danger coded from 1 (strongly agree) to 4 (strongly disagree).	1.8–4	3.7	3.5 ± 0.5
Social Danger Perception	Perceived presence of social incivilities and micro-crimes (e.g., illicit drug use, robberies, strange people) coded from 1 (strongly agree) to 4 (strongly disagree).	1–4	2.9	2.9 ± 0.7
Neighbourhood Relations	Perceived neighbourhood cohesion (e.g., stopping to talk to neighbours, exchanging favours with neighbours) coded from 1 (never) to 5 (everyday).	1.4–4.4	3.4	3.3 ± 0.7
**Mediating Variables**				
License for Independent Mobility	Number of independent activities the parent allows the child to do: cross roads, walk places, travel to and from school, go out after dark, cycle, public transit. Six items coded 0 (no) or 1 (yes).	0–6	3	3.3 ± 1.6
Roaming Allowance	How far the parent allows the child to go alone coded from 1 (not allowed) to 6 (more than 15-minute walk from home).	1–6	5	5.1 ± 1.1
**Dependent Variables**				
Territorial Range Area (km^2^) *	GPS-based convex hull area representing total space the child went without an adult in a seven-day period.	0–505	0.29	6.5 ± 50
Territorial Range Distance (km) *	GPS-based crow-fly distance representing the furthest the child went without an adult in a seven-day period.	0–32	0.84	1.7 ± 3.4

Note: N = number; SD = standard deviation; * N = 103 for territorial range area and territorial range distance.

**Table 4:** Path coefficients for territorial range area models (N = 105).

Variable	Total Effect		Model 1 Direct		Model 1 Indirect		Model 2 Direct		Model 2 Indirect	
	B (95% CI)	β	B (95% CI)	β	B (95% CI)	β	B (95% CI)	β	B (95% CI)	β
**Independent Variables**										
Destinations	−0.71 (−1.86, 0.43)	−0.16	−0.91 (−2.04, 0.21)	−0.21	0.19 (−0.13, 0.50)	0.04	−0.67 (−1.76, 0.42)	−0.15	-−0.05 (−0.40, 0.30)	−0.01
Recreation Facilities	0.16 (−1.29, 1.61)	0.27	0.21 (−1.19, 1.62)	0.04	−0.09 (−0.44, 0.26)	−0.02	0.39 (−0.99, 1.78)	0.07	−0.18 (−0.62, 0.26)	−0.03
Residential Density	0.02 (0.00, 0.03)	0.16	0.02 (0.00, 0.03)	0.15	0.00 (0.00, 0.00)	0.01	0.01 (−0.01, 0.03)	0.10	0.01 (0.00, 0.01)	0.06
Land-use Mix Access	−0.83 (−2.59, 0.94)	−0.09	−0.92 (−2.64, 0.79)	−0.01	0.10 (−0.33, 0.52)	0.01	−0.55 (−2.24, 1.14)	−0.06	−0.31 (−0.87, 0.26)	−0.03
Street Connectivity	0.26 (−1.03, 1.55)	0.04	0.20 (−1.05, 1.46)	0.03	0.09 (−0.22, 0.39)	0.01	−0.03 (−1.28, 1.22)	0.00	0.23 (−0.17, 0.64)	0.04
Walking Facilities	0.31 (−0.66, 1.28)	0.06	0.14 (−0.81, 1.09)	0.03	0.15 (−0.11, 0.41)	0.03	−0.05 (−1.00, 0.90)	−0.01	0.37 (0.00, 0.75) *	0.08
Aesthetics	0.15 (−1.04, 1.34)	0.03	0.16 (−0.99, 1.32)	0.03	−0.03 (−0.31, 0.25)	−0.01	0.15 (−0.98, 1.28)	0.03	0.03 (−0.33, 0.39)	0.01
Traffic Safety	1.03 (−0.46, 2.53)	0.14	1.30 (−0.17, 2.76)	0.18	−0.25 (−0.66, 0.16)	−0.03	0.97 (−0.46, 2.39)	0.13	0.03 (−0.42, 0.47)	0.00
Crime Safety	−0.31 (−1.61, 1.00)	−0.05	−0.73 (−2.04, 0.57)	−0.12	0.44 (−0.02, 0.90)	0.07	−0.79 (−2.07, 0.49)	−0.13	0.54 (0.04, 1.04) *	0.09
Social Danger Perception	−0.61 (−1.53, 0.31)	−0.13	−0.54 (−1.44, 0.36)	−0.12	−0.06 (−0.28, 0.15)	−0.01	−0.53 (−1.41, 0.36)	−0.11	−0.12 (−0.4, 0.15)	−0.03
Neighbourhood Relations	−0.12 (−0.95, 0.71)	−0.03	−0.21 (−1.02, 0.6)	−0.04	0.08 (−0.13, 0.29)	0.02	−0.55 (−1.38, 0.28)	−0.12	0.43 (0.06, 0.80) *	0.09
**Mediators**										
License for IM			0.56 (0.11, 1.01) *	0.26						
Roaming Allowance							1.21 (0.46, 1.96) ***	0.39		
**Covariates**										
Child age (ref: 10)										
11	1.74 (0.21, 3.27) *	0.23	1.18 (−0.37, 2.73)	0.16	0.54 (−0.03, 1.11)	0.07	0.93 (−0.62, 2.47)	0.12	0.82 (0.14, 1.51) *	0.11
12	2.95 (1.41, 4.49) ***	0.39	2.08 (0.42, 3.73) *	0.27	0.86 (0.08, 1.64) *	0.11	1.50 (−0.23, 3.23)	0.2	1.46 (0.45, 2.48) **	0.19
13	4.79 (3.20, 6.39) ***	0.62	3.43 (1.53, 5.33) ***	0.44	1.3 (0.2, 2.41) *	0.17	2.82 (0.86, 4.77) **	0.36	1.97 (0.66, 3.27) **	0.25
Child gender (ref: Female)										
Male	0.79 (−0.29, 1.87)	0.12	0.65 (−0.41, 1.7)	0.10	0.17 (−0.12, 0.46)	0.03	0.60 (−0.43, 1.63)	0.09	0.20 (−0.15, 0.54)	0.03
Income (ref: <$50,000)										
$50,000–$89,999	−2.27 (−4.4, −0.13) *	−0.28	−2.03 (−4.11, 0.05)	−0.25	−0.31 (−0.89, 0.27)	−0.04	−1.17 (−3.34, 0.99)	−0.15	−1 (−1.89, −0.11) *	−0.12
$90,000–$129,999	−2.26 (−4.25, −0.28) *	−0.31	−2.34 (−4.27, −0.41) *	−0.32	0.06 (−0.45, 0.56)	0.01	−2.01 (−3.92, −0.09) *	−0.27	−0.29 (−0.93, 0.34)	−0.04
≥$130,000	−1.20 (−3.18, 0.78)	−0.17	−1.24 (−3.16, 0.68)	−0.18	0.05 (−0.46, 0.56)	0.01	−1.04 (−2.94, 0.86)	−0.15	−0.18 (−0.8, 0.45)	−0.03

Note: * *p* < 0.05, ** *p* < 0.01, *** *p* < 0.001; B: unstandardized coefficient; β: standardized coefficient; CI: confidence interval; IM: independent mobility; Income: total household income. Model 1: Parental perceptions of neighbourhood environment on territorial range area, mediated by license for IM. Model 2: Parental perceptions of neighbourhood environment on territorial range area, mediated by roaming allowance.

**Table 5:** Path coefficients for territorial range distance models (N = 105).

Variable	Total Effect		Model 3 Direct		Model 3 Indirect		Model 4 Direct		Model 4 Indirect	
	B (95% CI)	β	B (95% CI)	β	B (95% CI)	β	B (95% CI)	β	B (95% CI)	β
**Independent Variables**										
Destinations	−0.44 (−1.02, 0.14)	−0.20	−0.52 (−1.10, 0.05)	−0.24	0.08 (−0.06, 0.22)	0.04	−0.42 (−0.98, 0.14)	−0.19	−0.02 (−0.19, 0.15)	−0.01
Recreation Facilities	0.05 (−0.69, 0.79)	0.02	0.07 (−0.66, 0.79)	0.02	−0.04 (−0.18, 0.11)	-0.01	0.16 (−0.55, 0.87)	0.05	−0.09 (−0.31, 0.13)	−0.03
Residential Density	0.01 (0.00, 0.02) *	0.22	0.01 (0.00, 0.02) *	0.21	0.00 (0.00, 0.00)	0.01	0.01 (0.00, 0.02)	0.16	0 .00 (0.00, 0.01)	0.05
Land-use Mix Access	−0.60 (−1.51, 0.30)	−0.13	−0.65 (−1.53, 0.24)	−0.14	0.04 (−0.14, 0.22)	0.01	−0.47 (−1.34, 0.40)	−0.10	−0.15 (−0.43, 0.13)	−0.03
Street Connectivity	0.13 (−0.53, 0.78)	0.04	0.10 (−0.55, 0.75)	0.03	0.04 (−0.09, 0.16)	0.01	−0.02 (−0.66, 0.62)	−0.01	0.11 (−0.09, 0.31)	0.04
Walking Facilities	0.13 (−0.36, 0.63)	0.05	0.06 (−0.43, 0.55)	0.03	0.06 (−0.05, 0.18)	0.02	−0.04 (−0.53, 0.45)	−0.02	0.18 (0.00, 0.37)	0.07
Aesthetics	0.31 (−0.30, 0.92)	0.10	0.32 (−0.28, 0.91)	0.11	−0.01 (−0.13, 0.11)	0.00	0.31 (−0.27, 0.89)	0.10	0.02 (−0.16, 0.19)	0.01
Traffic Safety	0.59 (−0.17, 1.35)	0.16	0.70 (−0.05, 1.46)	0.19	−0.10 (−0.29, 0.08)	-0.03	0.56 (−0.17, 1.29)	0.15	0.01 (−0.21, 0.23)	0.00
Crime Safety	−0.27 (−0.94, 0.39)	−0.09	−0.45 (−1.12, 0.23)	−0.14	0.18 (−0.04, 0.40)	0.06	−0.50 (−1.16, 0.15)	−0.16	0.26 (0.01, 0.52) *	0.09
Social Danger Perception	−0.29 (−0.76, 0.18)	−0.12	−0.26 (−0.73, 0.20)	−0.11	−0.03 (−0.12, 0.07)	-0.01	−0.25 (−0.70, 0.20)	−0.11	−0.06 (−0.20, 0.08)	−0.03
Neighbourhood Relations	−0.13 (−0.55, 0.30)	−0.05	−0.16 (−0.58, 0.25)	−0.07	0.03 (−0.06, 0.12)	0.01	−0.34 (−0.76, 0.09)	−0.14	0.21 (0.03, 0.40) *	0.09
**Mediators**										
License for IM			0.23 (0, 0.47) *	0.22						
Roaming Allowance							0.59 (0.21, 0.98) ***	0.37		
**Covariates**										
Child age (ref: 10)										
11	0.73 (−0.05, 1.51)	0.19	0.50 (−0.30, 1.30)	0.13	0.23 (−0.05, 0.50)	0.06	0.33 (−0.46, 1.12)	0.09	0.40 (0.06, 0.75) *	0.11
12	1.46 (0.67, 2.24) ***	0.37	1.09 (0.24, 1.94) *	0.28	0.36 (−0.03, 0.75)	0.09	0.74 (−0.15, 1.63)	0.19	0.72 (0.20, 1.24) **	0.18
13	2.33 (1.52, 3.14) ***	0.59	1.76 (0.78, 2.74) ***	0.44	0.54 (−0.01, 1.10)	0.14	1.36 (0.36, 2.36) **	0.34	0.96 (0.30, 1.63) **	0.24
Child gender (ref: Female)										
Male	0.32 (−0.23, 0.87)	0.09	0.25 (−0.29, 0.80)	0.08	0.07 (−0.06, 0.20)	0.02	0.22 (−0.31, 0.75)	0.07	0.10 (−0.07, 0.27)	0.03
Income (ref: <$50,000)										
$50,000–$89,999	−1.02 (−2.11, 0.07)	−0.25	−0.92 (−2.00, 0.16)	−0.23	−0.13 (−0.38, 0.13)	-0.03	−0.48 (−1.59, 0.63)	−0.12	−0.49 (−0.94, −0.04) *	−0.12
$90,000–$129,999	−1.26 (−2.28, −0.25) *	−0.34	−1.31 (−2.30, −0.31) *	−0.35	0.03 (−0.19, 0.24)	0.01	−1.14 (−2.12, −0.16) *	−0.30	−0.14 (−0.45, 0.17)	−0.04
≥$130,000	−0.52 (−1.53, 0.49)	−0.15	−0.54 (−1.53, 0.45)	−0.15	0.02 (-0.19, 0.24)	0.01	−0.44 (−1.41, 0.54)	−0.13	−0.09 (−0.40, 0.22)	−0.03

Note: * *p* < 0.05, ** *p* < 0.01, *** *p* < 0.001; B: unstandardized coefficient; β: standardized coefficient; CI: confidence interval; IM: independent mobility; Income: total household income. Model 3: Parental perceptions of neighbourhood environment on territorial range distance, mediated by license for IM. Model 4: Parental perceptions of neighbourhood environment on territorial range distance, mediated by roaming allowance.
